# Subjective assessment underestimates surgical risk: On the potential benefits of cardiopulmonary exercise testing for open thoracoabdominal repair

**DOI:** 10.1111/jocs.16574

**Published:** 2022-04-29

**Authors:** Damian M. Bailey, Claire L. Halligan, Richard G. Davies, Anthony Funnell, Ian R. Appadurai, George A. Rose, Lara Rimmer, Matti Jubouri, Joseph S. Coselli, Ian M. Williams, Mohamad Bashir

**Affiliations:** ^1^ Neurovascular Research Laboratory, School of Health, Sport and Professional Practice, Faculty of Life Sciences and Education University of South Wales Pontypridd UK; ^2^ Department of Anaesthesia Wrexham Maelor Hospital Wrexham UK; ^3^ Department of Anaesthetics University Hospital of Wales Cardiff UK; ^4^ Department of Anaesthetics, Princess of Wales Hospital, Velindre University NHS Trust Health Education & Improvement Board Wales (HEIW) Wales UK; ^5^ North West School of Surgery Health Education England North West Manchester UK; ^6^ University of York York UK; ^7^ Division of Cardiothoracic Surgery, Michael E. DeBakey Department of Surgery Baylor College of Medicine Houston USA; ^8^ Department of Cardiovascular Surgery Texas Heart Institute Houston Texas USA; ^9^ Department of Cardiovascular Surgery CHI St Luke's Health‐Baylor St Luke's Medical Center Houston Texas USA; ^10^ Department of Surgery University Hospital of Wales Cardiff UK

**Keywords:** aneurysm, aorta, cardiopulmonary exercise testing, fitness, frailty, thoracoabdominal

## Abstract

**Background:**

Initial clinical evaluation (ICE) is traditionally considered a useful screening tool to identify frail patients during the preoperative assessment. However, emerging evidence supports the more objective assessment of cardiorespiratory fitness (CRF) via cardiopulmonary exercise testing (CPET) to improve surgical risk stratification. Herein, we compared both subjective and objective assessment approaches to highlight the interpretive idiosyncrasies.

**Methods:**

As part of routine preoperative patient contact, patients scheduled for major surgery were prospectively “eyeballed” (ICE) by two experienced clinicians before more detailed history taking that also included the American Society of Anesthesiologists score classification. Each patient was subjectively judged to be either “frail” or “not frail” by ICE and “fit” or “unfit” from a thorough review of the medical notes. Subjective data were compared against the more objective validated assessment of postoperative outcomes using established CPET “cut‐off” metrics incorporating peak pulmonary oxygen uptake, V̇O_2PEAK_ at the anaerobic threshold (V̇O_2_‐AT), and ventilatory equivalent for carbon dioxide that collectively informed risk stratification. These data were retrospectively extracted from a single‐center prospective National Health Service database. Data were analyzed using the Chi‐square automatic interaction detection decision tree method.

**Results:**

A total of 127 patients were examined that comprised 58% male and 42% female patients aged 69 ± 10 years with a body mass index of 29 ± 7 kg/m^2^. Patients were poorly conditioned with a V̇O_2PEAK_ almost 20% lower than predicted for age, sex‐matched healthy controls with 35% exhibiting a V̇O_2_‐AT < 11 ml/kg/min. Disagreement existed between the subjective assessments of risk with ∼34% of patients classified as not frail on ICE were considered unfit by notes review (*p* < .0001). Furthermore, ∼35% of patients considered not frail on ICE and ∼31% of patients considered fit by notes review exhibited a V̇O_2_‐AT < 11 ml/kg/min, and of these, ∼28% and ∼19% were classified as intermediate to high risk.

**Conclusions:**

These findings highlight the interpretive limitations associated with the subjective assessment of patient frailty with surgical risk classification underestimated in up to a third of patients compared to the validated assessment of CRF. They reinforce the benefits of a more objective and integrated approach offered by CPET that may help us to improve perioperative risk assessment and better direct critical care provision in patients scheduled for “high‐stakes” surgery including open thoracoabdominal aortic aneurysm repair.

**Abbreviations**
ASA: American Society of Anesthesiologists score
CPET: cardiopulmonary exercise testing
ICE: initial clinical evaluation
V̇_E_/V̇CO_2_
: ventilatory equivalent for carbon dioxide
V̇O_2_‐AT: pulmonary oxygen uptake at the anaerobic threshold
V̇O_2PEAK_
: peak oxygen uptake

## 1 BACKGROUND

Traditionally, the assessment of fitness for surgery involves a surgeon's subjective judgment on whether a patient is sufficiently conditioned to undergo the proposed procedure. Valid and reliable assessment of a person's functional capacity is thus considered an important component of preoperative evaluation.^1^ The initial clinical evaluation (ICE) can be a useful screening tool to identify frail patients in the preoperative assessment, despite limited research to validate the implementation. “Frailty” identifies those patients with a diminished capacity to compensate adequately for external stressors who are at greater risk of adverse outcomes including a prolonged hospital stay, institutionalization, worsening disability, and even death.^2^, ^3^ It is important to recognize diminished capacity in patients before surgery given that they are less likely to survive or return to functional status following the physiological insult of surgery compared to their fitter, more resilient counterparts.^4^

ICE almost inextricably requires a clinician to make a rapid decision concerning the fitness for an operation based on little more than external appearances. In contrast, preoperative cardiopulmonary exercise testing (CPET) enhances the integrated risk assessment by providing a more objective measure to establish if a patient has adequate cardiorespiratory fitness (CRF) to tolerate major surgery. In support, CPET has gained popularity as part of the routine preoperative diagnostic assessment and its predictive value in relation to mid‐ and long‐term survival in patients undergoing elective open surgical abdominal aortic aneurysm (AAA) repair is well established including its ability to forecast postoperative morbidity.^5^, ^6^, ^7^

**FIGURE 1:**
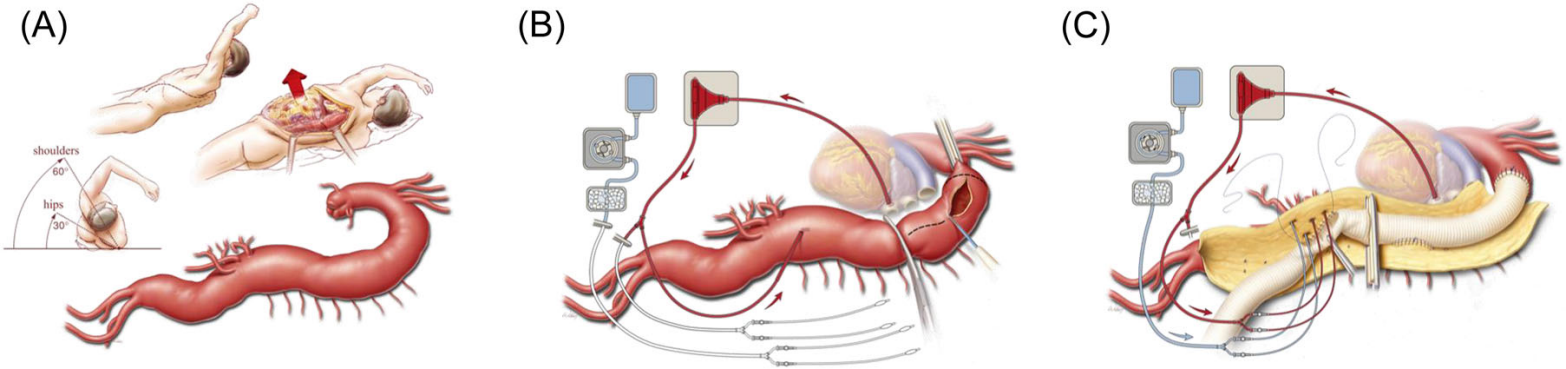
Anatomical aspects and surgical approach to extent II thoracoabdominal repair. (A) The chest is entered through the sixth intercostal space. Left medial visceral rotation and circumferential division of the diaphragm enable exposure of the entire thoracoabdominal aorta. (B) Left heart bypass (LHB) is commonly used to offload the heart from stressors of aortic surgery; LHB is initiated by placing a cannula in the left atrium via a left inferior pulmonary venotomy and then connecting it to the drainage line of the LHB circuit. After initiation, the proximal aortic clamp is placed. The distal aortic clamp is placed across the mid‐descending thoracic aorta. The aortic segment between the two clamps is opened longitudinally using electrocautery. A stand‐alone circuit to provide cold renal perfusion is prepared for later use. (C) Following completion of the proximal anastomosis, the aorta is opened longitudinally to the aortic bifurcation. Crucial intercostal and lumbar arteries are reattached. Cold renal perfusion and selective visceral perfusion are provided to protect the visceral organs.

This is especially relevant for open thoracoabdominal aortic aneurysm (TAAA) surgery, given that it requires careful selection of patients who will be suitable to undergo extensive surgery and lengthy postoperative recovery (Figure 1). Predictive risk models have shown that multi‐system impairment is related to negative operative outcomes predisposing to longer recovery times and increased risk of short‐ and long‐term mortality and morbidity.^8^ Lung disease, older age, female sex, New York Heart Association's moderate (III) or severe (IV) classifications, and reduced left ventricular ejection fraction have been identified as independent risk factors for patients undergoing proximal aortic repair.^9^ However, there is no singular metric with the capacity to accurately predict clinical outcome.^2^

Thus, it is suspected that patients with poor CRF are especially vulnerable when faced with the enhanced metabolic demands posed by open TAAA repair and have an unmet need to better guide patient evaluation, risk, and clearance for surgery. In the coming years when both open and endovascular options for thoracoabdominal aortic repair are widely available, there will no doubt be a need to objectively evaluate each patient to identify the ideal method of surgical repair.

To that end, the present study sought to compare subjective ICE (eyeballing) by experienced clinicians against the more objective validated preoperative assessment using formalized CPET metrics for patients undergoing major elective surgery. We hypothesized that subjective assessment would underestimate a patient's “true” surgical risk, highlighting the benefits of a more integrated objective approach that has direct relevance for patients scheduled for open TAAA repair.

## 2 METHODS

### 2.1 Ethical approval

The Cardiff and Vale University Health Board (15/AIC/6352) approved the retrospective analysis of an anonymized database and thus patient consent was waived. All procedures were carried out in accordance with the Declaration of Helsinki of the World Medical Association.^10^

### 2.2 Design

Clinical data were extracted from a single‐center (University Hospital of Wales, UK) prospective National Health Service database for the purposes of improving perioperative outcomes in patients scheduled for elective major intra‐abdominal surgery over a 12‐month period. Data points were captured using a variety of methods, including medical record abstraction and formal data collection (below).

### 2.3 Clinical assessments

#### 2.3.1 Demographics

Patient data were gathered from medical notes and recorded by the clinician conducting CPET and comprised stature, body mass, derivation of body mass index (BMI), and closed‐loop flow spirometry.

#### 2.3.2 Subjective assessment

As part of the routine evaluation of patients before surgery, patients were clinically assessed by two experienced clinical consultants to determine frailty and fitness for surgery. The clinical evaluation included the detailed collection of a patient‐specific medical history. This clinical determination aimed to answer the question “Is this patient attending for clinical assessment today fit enough for the proposed surgical procedure?” Each patient was judged to be either “frail” or “not frail” after the initial meeting, and this was supplemented with “fit” or “not fit” from a careful review of their medical notes. Patients were also graded according to the American Society of Anesthesiologists (ASA) grading criteria^11^ whereby a healthy patient is ASA I, a patient with the mild systemic disease is ASA II, a patient with the severe systemic disease is ASA III, ASA IV refers to a patient with life‐threatening severe systemic disease and ASA V to a moribund patient.

#### 2.3.3 Objective assessment

2.3.3.1

*CPET*: Preoperative CPET was conducted using an electromagnetically braked cycle ergometer (Lode) and a Medgraphics Ultima metabolic cart (MedGraphics^TM^) as previously outlined by our group.^7^, ^12^, ^13^ Briefly, calibration was undertaken in accordance with the manufacturer's guidelines using a 3‐L volume syringe (Hans Rudolph) and reference calibration gases. During data collection, the middle five of seven breaths were averaged. An exercise protocol was employed requiring patients to cycle at 60 rpm for 3 min in an unloaded freewheeling state followed by a progressively ramped period of exercise (5–15 W/min based on mass, stature, age, and sex) to volitional or symptom‐limited termination, followed by 3 min recovery.^14^ Medgraphics Breeze^TM^ software automatically determined V̇O_2PEAK_ (defined as the highest V̇O_2_ during the final 30 s of exercise reported), the slope of the relationship between pulmonary ventilation and carbon dioxide output (V̇_E_/V̇CO_2_) and oxygen uptake efficiency slope (OUES). Pulmonary oxygen uptake at the anaerobic threshold (V̇O_2_‐AT) was manually interpreted by an experienced clinician using the V‐slope method,^15^ supported by V̇_E_/V̇CO_2_‐AT, and V̇_E_/V̇O_2_‐AT.

2.3.3.2

*Risk classification*: Each patient was classified with a V̇O_2_‐AT below (<) or above (>) 11 ml O_2_/kg/min based on the seminal works of Weber and Janicki^16^ and Older et al.^17^ We further differentiated between low, intermediate, and high risk according to the following criteria: *Low risk*: V̇O_2_‐AT ≥ 11 ml/kg/min; *intermediate risk*: one of: V̇O_2_‐AT 8–10.9 ml/kg/min, V̇_E_/V̇CO_2_‐AT > 34, history of ischemic heart disease (IHD); *high risk*: V̇O_2_‐AT < 8 ml/kg/min or ≥two of: V̇_E_/V̇CO_2_‐AT > 34, V̇O_2_‐AT < 11 ml/kg/min, history of IHD.

### 2.4 Statistical analyses

Statistical analyses were undertaken using IBM SPSS Statistics for Windows (Version 28.0; IBM). Continuous variables are reported as mean ± standard deviation. Categorical variables are reported as frequencies with percentages. Categorical comparisons were conducted using χ^2^ tests and χ^2^ automatic interaction detection decision tree method.

## 3 RESULTS

### 3.1 Patient characteristics

Table 1 summarizes patient characteristics including demographics and cardiopulmonary performance (spirometry and CPET) with a total of 127 patients examined. Thirty‐nine patients (31%) were classified as obese with 56 (44%) overweight. As anticipated, these patients were poorly conditioned with a V̇O_2PEAK_ that was on average almost 20% lower than predicted for age, sex‐matched healthy controls with 45 patients (35%) exhibiting a V̇O_2_‐AT < 11 ml/kg/min. V̇O_2_‐AT could not be determined in 18 patients (14%).

**TABLE 1:** Patient characteristics

*Demographics*	
Sample size (*n*)	127
Male (*n*/%):Female (*n*/%)	74/58:53/42
Age (y)	69 ± 10
BMI (kg/m^2^)	29 ± 7
*Spirometry*	
FVC (% predicted)	95 ± 19
FEV_1_ (% predicted)	92 ± 22
FEV_1_/FVC (% predicted)	73 ± 9
*CPET metrics*	
Peak workload (W)	95 ± 43
Peak workload (% predicted)	85 ± 29
V̇O_2PEAK_ (ml/kg/min)	17.8 ± 5.2
V̇O_2PEAK_ (% predicted)	81 ± 20
V̇_E_/V̇CO_2_ slope (AU)	34 ± 6
OUES ([ml/min O_2_]/[L/min *V̇*_E_])	1729 ± 490
V̇O_2_‐AT > 11 ml/kg/min (*n*/%)	64/50
V̇O_2_‐AT < 11 ml/kg/min (*n*/%)	45/35
V̇O_2_‐AT indeterminate (*n*/%)	18/14

*Note*: Values are mean ± SD.

Abbreviations: BMI, body mass index; CPET, cardiopulmonary exercise testing; FEV_1_, forced expiratory volume in 1 s; FVC, forced vital capacity; OUES, oxygen uptake efficiency slope; V̇_E_/V̇CO_2_, ventilatory equivalent for carbon dioxide; V̇O_2‐AT_, pulmonary oxygen uptake at the anaerobic threshold; V̇O_2PEAK_, peak pulmonary oxygen uptake.

### 3.2 Clinical risk assessments

**FIGURE 2:**
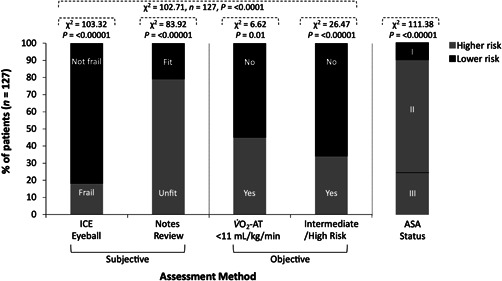
Differences in clinical risk classification according to patient assessment method. ASA, American Society of Anesthesiologists score (ASA I, normal healthy, ASA II, mild systemic disease, ASA III, severe systemic disease); ICE, initial clinical evaluation; V̇O_2_‐AT, pulmonary oxygen uptake at the anaerobic threshold during cardiopulmonary exercise testing.

**FIGURE 3:**
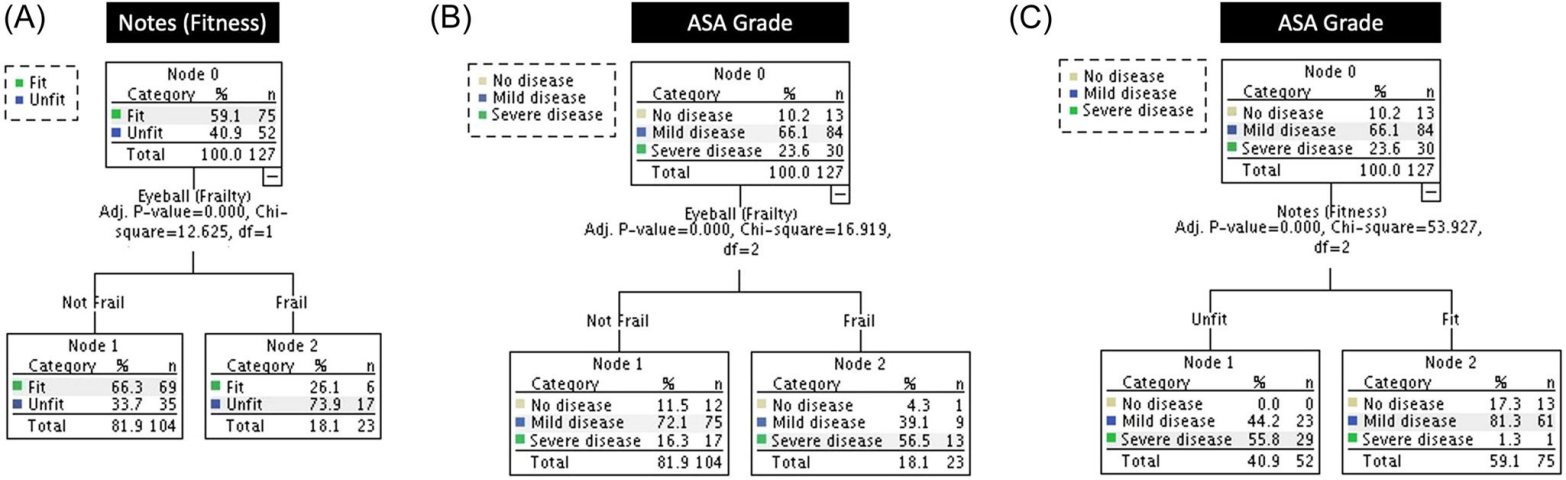
Comparison between different subjective methods of patient risk assessment. ASA, American Society of Anesthesiologists score (ASA I, normal healthy, ASA II, mild systemic disease, ASA III, severe systemic disease).

Figure 2 illustrates the patient distribution of clinical risk classification according to the assessment method providing a visual of the (dis) agreements observed complemented by the Chi‐square automatic interaction detection decision tree method analyses summarized in Figures 2 and 3.

#### 3.2.1 Subjective

There was clear disagreement between the subjective assessments of risk (ICE‐Eyeball (Frailty) compared to Notes Review (Fitness) with ∼34% of patients classified as not frail and considered unfit by notes review (Figure 3A). Equally, ∼88% of patients considered not frail and ∼82% of patients considered fit by ICE (Figure 3B) and notes review (Figure 3C), respectively, were classified ASA Grade II–III (mild to severe disease).

#### 3.2.2 Objective

**FIGURE 4:**
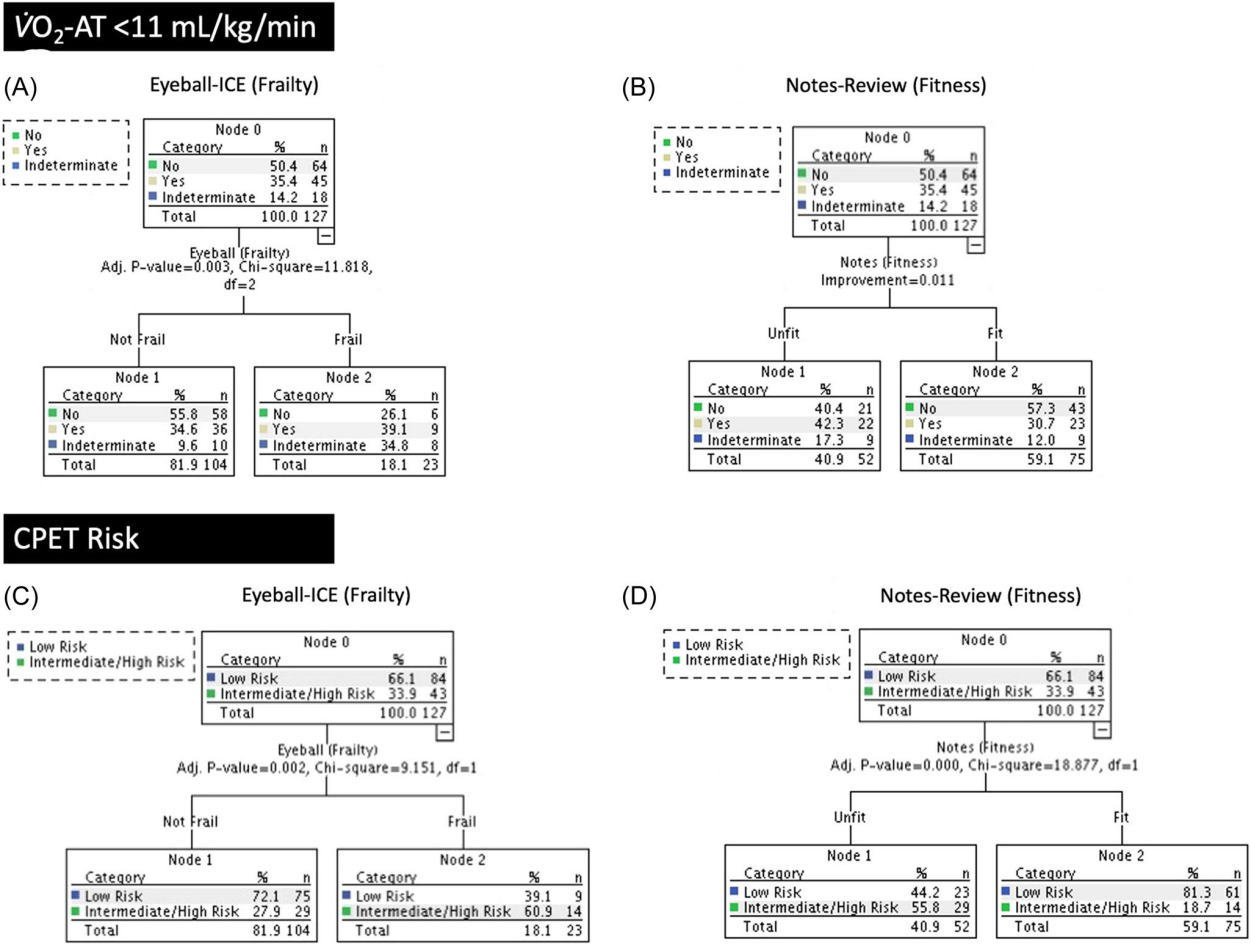
Comparison of subjective assessment of patient risk (initial clinical evaluation [ICE] of frailty and notes‐review of fitness) against risk defined by objective cardiopulmonary exercise testing (CPET) metrics. See Section 2 for the definition of low, intermediate, and high risk. V̇O_2_‐AT, pulmonary oxygen uptake at the anaerobic threshold.

Subjective assessments generally underestimated patient risk compared to objective CPET criteria (V̇O_2_‐AT < 11 ml/kg/min and intermediate‐to‐high risk). Indeed, ∼35% of patients considered not frail on ICE and ∼31% of patients considered fit by notes review exhibited a V̇O_2_‐AT < 11 ml/kg/min (Figure 4A,B). Of these, ∼28% and ∼19% (not frail and fit patients, respectively) were classified as an intermediate‐to‐high risk by CPET criteria (Figure 4C,D).

## 4 DISCUSSION

By comparing subjective clinical evaluation of a patient's risk by experienced clinicians against the more objective validated assessment of postoperative outcomes via CPET, the present study has identified two important findings. First, we identified clear disagreement between subjective assessments of risk with a third of patients classified not frail considered unfit by notes review and almost 9 out of 10 patients suffering from mild to severe disease by ASA classification. Second, and consistent with our original hypothesis, subjective assessment underestimated surgical risk in up to a third of patients. Collectively, these findings highlight the benefits of a more objective and integrated approach offered by CPET that may help improve perioperative risk assessment and direct care provision in patients scheduled for ‘high‐stakes' open TAAA repair.

### 4.1 Surgical demands

Surgery is the third largest cause of death after IHD and stroke accounting for almost 8% of all deaths globally.^18^ Given the aging population and projected burden of vascular arterial occlusive/aneurysmal disease, surgery remains a major concern for healthcare providers. Importantly, the “high risk” surgical patient accounts for 13% of cases yet contributes to a disproportionate >80% of all postoperative deaths and complications.^19^ This is especially the case for TAAA patients given the extensive repair required and prolonged recovery time with increasing interest direct towards the “gold‐standard” assessment of CRF via CPET to provide more objective insight into surgical risk stratification.

An adequate, although presently undefined CRF conferring improved physiological reserve is required in order for a patient to tolerate extensive open TAAA repair, given that single lung ventilation is obligatory to expose the thoracic aorta following the collapse of the left lung (Figure 1). Acceptable preoperative spirometry assessment of the pulmonary circulation may consist of a forced expiratory volume in 1 s > 1 L and arterial partial pressure of carbon dioxide <45 mmHg.^19^ Postoperative pulmonary complications and reintubation rates of up to 15% in the highest volume centers indicate that this remains a major cause of morbidity following TAAA surgery.^20^ Pulmonary complications occur in up to 36% of patients and any adverse lung function tests preoperatively, highlighted through spirometry and arterial blood gas analysis, may be advised to undergo a regime including physical exercise, spirometry training, and bronchodilator therapy.^21^ Other factors reducing prolonged ventilator support included preservation of the central tendon of the diaphragm by circumferential division and avoidance of excessive blood products.^22^ Postoperatively, adequate pulmonary function is essential for perioperative survival as all patients will be intubated in the immediate and extensive postoperative recovery phase.

### 4.2 Patient comorbidities

Importantly, many patients undergoing TAAA repair will have pre‐existing coronary artery disease (CAD) and associated risk factors.^20^ Significant (but possibly silent) cardiac disease may reduce patient tolerance of thoracic aortic cross‐clamping, an obligatory procedure that immediately increases afterload, and left ventricular stress, upon the heart.^21^ Oxygen deprivation in proximal tissues and sympathoadrenal discharge constricts arterioles and is typically accompanied by arteriovenous shunting. While acute (CPET) exercise may not replicate the profound physiological challenges imposed by cross‐clamping, assessing the patient's body under “simulated” (physical) stress and corresponding systemic response to microcirculatory hypoxemia may determine how well systemic tissue perfusion adapts to the surgical insult.

Furthermore, there is mounting evidence that the risk of developing spinal cord ischemia is increased by up to 80% in those with CAD.^23^ Identification of disease may not negate surgery but may lead to optimization by coronary artery stenting or instigating antiplatelet therapy before any planned procedure.^21^, ^24^ Connective tissue disorders represent an additional major risk factor for thoracic aortic disease with up to 20% of patients expressing at least one “high‐risk” gene.^25^ Marfan syndrome (MS) is one such genetic disorder and typically presents in younger patients.^26^ Giske et al.^27^ focused on pulmonary function and rehabilitation in patients with MS and found that *V̇*O_2PEAK_ was 30% and 50% lower in females and males respectively, compared to healthy (non‐MS) controls.

### 4.3 TAAA surgery and CPET

While there is clear justification for the integration of CPET into perioperative risk assessment for open TAAA, there are surprisingly few studies in the published literature. Hornsby et al.^28^ used CPET postoperatively only to assess exercise tolerance following open TAAA or type A dissection repair. CPET was analyzed retrospectively or performed 3 months following open repair and identified that (median) V̇O_2PEAK_ was reduced by 36% after type A aortic dissection repair. This highlights the critical increase in metabolic demand driven by the need to increase vascular O_2_ delivery to support the additional cellular bioenergetic demands incurred by surgery to ensure a successful recovery.^29^ If the patient is unable to fulfill this metabolic demand (i.e., CRF is inadequate), the physiological “insult” posed by TAAA surgery can subsequently lead to O_2_ debt that can overwhelm the patient and result in organ failure and death.^30^

In the present study, we chose to differentiate between those patients with and without “adequate” CRF based on the “cut‐off” metrics originally established by Weber and Janicki^16^ in heart failure patients and later implemented by Older et al.^17^ specifically V̇O_2_‐AT < (unfit) or > (fit) 11 ml/kg/min. Older et al.^17^ identified an 18% mortality rate in elderly surgical patients considered unfit by this threshold compared to 0.8% in fit patients. We further categorized patients based on CPET risk through the additional implementation of complementary biomarkers including V̇O_2_ peak <15 ml/kg/min and V̇_E_/V̇CO_2_‐AT > 34 given their combined ability to distinguish the “at‐risk” patient and better predict postoperative survival following AAA surgery.^31^ However, it is important to emphasize that ongoing research continues to better define threshold metrics to further optimize risk prediction models and this is especially relevant for TAAA patients given the magnitude of the surgical “hit” encountered. Furthermore, CRF (and corresponding risk) stratification needs to be based not on a single binary cut‐off but rather on a range of values for any given dynamic CPET metric given the inherent (and extensive) biological variation^13^ and this remains to be established for the “high‐stakes” TAAA patient.

Importantly and in stark contrast to the present study, none of these researchers have reported the clinician's initial views before surgery. There are understandable if not unavoidable limitations to what a clinician might gain from the very first review of a patient, often without a thorough knowledge of past medical history. Initial information is oftentimes dictated by loose “impressions” of cognitive function, body habitus, strength of a handshake, and general nutritional status.^32^ Our findings highlight that ICE is indeed unreliable compared to CPET metrics with the danger of underestimating patient risk. This has implications when determining the appropriate level of postoperative care after TAAA surgery notwithstanding the potential for medico‐legal complications.

Clinical assessment from the end of the bed will undoubtedly benefit from more comprehensive physiological testing. This is particularly the case for increasing numbers of patients with TAAA who are considered for endovascular rather than open surgery.^33^ It is likely that future treatment plans will incorporate both open and endovascular approaches for intervention and this may even be incorporated in a staged manner.^34^

## 5 CONCLUSION

These findings highlight the interpretive limitations associated with the subjective assessment of patient frailty with surgical risk classification underestimated in up to a third of patients compared to the more objective validated assessment of postoperative outcomes via CPET‐derived CRF. For “high‐stakes” open TAAA surgery, the integration of CPET can improve perioperative risk assessment though further research is required to identify “lower limits” of CRF below which operative intervention may be considered prohibitively risky. Surgeons also need to consider (preoperative) exercise training as a modifiable component of multimodal prehabilitation strategies with the potential to augment CRF, reduce surgical risk and thus improve outcomes.

## AUTHOR CONTRIBUTIONS

All authors read and approved the final manuscript.

## CONFLICTS OF INTEREST

Prof. Damian M. Bailey is a member of the National Cardiovascular Research Network (Wales), European Assiociation for Cardiovascular Prevention and Rehabilitation and European Society of Cardiology (Atherosclerosis and Vascular Biology). Dr. Joseph S. Coselli participates in clinical trials with and/or consults for Terumo Aortic, Medtronic, and W. L. Gore & Associates, and receives royalties and grant support from Terumo Aortic. The remaining authors declare no conflicts of interest.
